# GxP-Ready Single-Cell RNA-seq and Spatial Transcriptomics End-to-End Pipeline for Clinical Research

**DOI:** 10.34133/csbj.0164

**Published:** 2026-07-14

**Authors:** Amaya Zaratiegui, Timothy Burfield, Helle Rus Povlsen, Martín E. García Solá, Adrian Czaban, Keng Soh, Vivek Das

**Affiliations:** ^1^ Novo Nordisk A/S, Søborg, Denmark.; ^2^ ZS Associates, ZS Discovery, Buenos Aires, Argentina.

## Abstract

Single-cell/nucleus RNA-sequencing and Spatial Transcriptomics are powerful tools for investigating cellular heterogeneity and tissue architecture that have deepened our disease understanding. Their broader adoption in clinical and regulated settings, however, is hindered by regulatory requirements related to data integrity, regulatory compliance, reproducibility, and scalability. To address this gap, we developed NNclinSSOAP (Novo Nordisk Clinical Single-cell Spatial Omics Analytical Pipeline)—a modular, GxP-ready end-to-end computational pipeline that combines established single-cell workflows with a new Nextflow pipeline for Spatial Transcriptomics. NNclinSSOAP transforms RNA sequencing and Xenium spatial data into integrated, annotated single-cell objects and spatially resolved tissue maps. Designed to support mechanistic studies and clinical endpoint generation, it enables traceable and reproducible processing of large-scale datasets, scalable for use in HPC environments. Here, we provide a step-by-step demo case for using NNclinSSOAP that can be executed within 1.5 h on a standard laptop. All code and data are available open-source.

## Introduction

Single-cell/single-nucleus RNA sequencing (sc/snRNA-seq) has revolutionized biological research by enabling the study of gene expression at the resolution of individual cells and nuclei. The technology allows for the identification of cell types, the characterization of cellular heterogeneity within tissues, underlying cell–cell communication, and the understanding of complex biological processes at an unprecedented level of detail [1]. This granularity can uncover rare cell populations, specific gene expression profiles, and dynamic changes over time [2]. However, it inherently lacks spatial [3] information, which is critical for understanding the tissue architecture and the local microenvironment in which the cells reside [4]. Conversely, Spatial Transcriptomics retains high-resolution spatial information but often lacks the single-cell resolution necessary to discern the complexity of cellular interactions and the interplay of different cell types within tissues [5].

The multimodal omics approach of combining scRNA-seq and Spatial Transcriptomics addresses their respective limitations and provides a more comprehensive overview of cellular mechanisms in tissue contexts. This enables cellular identity and spatial organization to be established concurrently, which can aid in revealing the mechanisms of action of drugs and understanding disease pathology [6–8].

The use of these technologies and the large data handling requirements that come with them pose challenges for use in clinical settings. Ideally, such data need to be stored and handled in a Good Experimental Practice (GxP)-compliant way and compute resources need to be large enough to handle the data volumes.

This paper addresses these critical challenges, describing the implementation and usage instructions for NNclinSSOAP (Novo Nordisk Clinical Single-cell Spatial Omics Analytical Pipeline)—a GxP-ready pipeline for running sc/snRNA-seq and Spatial Transcriptomics together. Our approach ensures data integrity and facilitates the translation of findings into robust and reliable conclusions for downstream applications, contributing to the standardization and quality control necessary for wider application of sc/snRNA-seq and Spatial Transcriptomics in regulated environments and clinical trial settings. Unlike applications aimed primarily at interactive exploration, NNclinSSOAP is designed as an open-source, configurable pipeline that users can adapt to their own study designs, analysis needs, and compute environments.

## Pipeline Overview

The development of this paper was motivated by the need for a combined sc/snRNA-seq and Spatial Transcriptomics workflow in a clinical research and trial endpoint analysis context. While single-cell and Spatial Transcriptomics technologies have become widely used for tissue and disease understanding for hypothesis generation, their application in regulated environments such as clinical trials presents specific challenges related to GxP requirements. Existing workflows, although effective in research settings, often do not address these requirements in a systematic way.

To meet these needs, we designed an end-to-end pipeline that integrates established methods and best practices from the broader community, with adaptations that ensure GxP-readiness and scalability. It was developed to ingest, process, and analyze sc/snRNA-seq and spatial data to generate clinical endpoints and run predefined analysis as per trial analysis plans, while providing a transparent and auditable framework suitable for clinical studies. Because of the number of samples set to be collected from multiple omics modalities, it was clear that the workflow would need to handle large data volumes and support execution in a high-performance computing (HPC) environment. To address these points, we adopted a modular, containerized architecture, enabling compliant storage and processing environments to be connected to the NNclinSSOAP pipeline.

**Fig. 1.**
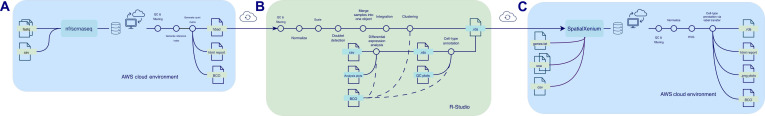
Overview of how the 3 main components of NNclinSSOAP are connected, with their respective inputs, processing steps, outputs, and compute environments. (A) sc/snRNA-seq workflow. Processes fastq data, outputting, among others, h5ad files together with a multi-qc report and a BCO. The main processing steps include Quality control, Generate reference index, and Generate count matrix. (B) R library/scripts workflow. Processes the h5ad file from (A), outputting an annotated Seurat RDS object. The main processing steps include Quality control, Doublet detection, Multiple sample integration, Seurat object generation, Differential expression analysis, and Cell-type annotation. (C) SpatialXenium workflow. Processes raw Xenium data, outputting Spatial Transcriptomics analyses together with a report and a BCO, optionally taking the RDS object from the previous step to add cell-type labels to the analyses. The main processing steps include Quality control, Data normalization, Dimensionality reduction and clustering, Cell label transfer, and Results visualization.

The development process was iterative and collaborative, involving and building on cross-departmental expertise in computational biology, cloud engineering, statistics, and regulatory science. To address diverse data types and evolving analytical requirements, NNclinSSOAP was designed in a modular fashion that allows for straightforward adaptation to new datasets, data modalities, and requirements, providing flexibility and scalability for future research requirements and analyses. It is composed of 3 main components, connected as illustrated in Fig. 1 and described below:

1. sc/snRNA-seq workflow: Handles the preprocessing, alignment, and quantification of single-nucleus RNA-seq data, leveraging nf-core/scrnaseq [9] for standardized and scalable data processing.
2. R library/scripts: Enables advanced postprocessing, quality control, annotation, and statistical analyses of the output from the sc/snRNA-seq pipelines, ensuring robust downstream interpretation.
3. SpatialXenium: A new Nextflow pipeline developed by us, designed to analyze multimodal data from the Spatial Transcriptomics platform Xenium [10], and optimized to run on HPCs ensuring efficient handling of large datasets. It ingests spatially resolved gene expression data and performs quality control, normalization, clustering, and spatial niche construction. The pipeline outputs processed Seurat objects (RDS), cluster/niche compositions, top marker genes, and visualizations (Principal Component Analysis [PCA], Uniform Manifold Approximation and Projection [UMAP], and spatial plots). Optionally, it performs label transfer, adding cell-type annotation to the visualizations. The submodules that make up SpatialXenium are described in further detail in Box 1.

Box 1. Brief introduction to the Spatial Transcriptomics processing steps (submodules)

1. **SEURAT_XENIUM**

• Orchestrates reading/validating data, quality control, filtering, dimensionality reduction, clustering, and saving results.
• Can optionally produce a generically named output RDS if --generic_name is set to “TRUE”.
Key functions

• *open_seurat()*: Loads Xenium data into Seurat, merging QC metadata.
• *plot_qc()*: Generates QC plots (e.g., read count distributions and gene feature counts).
• *filter_genes()*: Filters genes based on average quality value (QV) and read count thresholds.
• *run_seurat()*: Normalizes data, identifies variable features, and runs PCA/UMAP/clustering.
• *get_cell_markers()*: Finds marker genes for each cluster.
• *plot_processed_data()*: Visualizes PCA, feature distributions, and UMAP.

2. **LABEL_TRANSFER**

• Reads the query Seurat RDS, reference RDS, and label column parameters.
• Calls *transfer_labels()* to annotate the Xenium data.
• Supports a --future_mem_limit parameter to set memory usage for parallel operations.
• Outputs labeled Seurat RDS and prediction files.
Key functions

• *create_bygene_df()*: Summarizes gene counts across Seurat assays.
• *transfer_labels()*: Loads a reference RDS (single-cell data), ensuring matching label columns and overlapping features.
• Fits a linear model (query vs. reference gene counts), calculates residuals, and identifies anchors.
• Performs label transfer via *TransferData()*, generating predicted labels and probabilities.
• Filters low-probability cells (“Ambiguous”) and updates Seurat metadata.

3. **PLOT_FEATURE**

• Loads the Seurat object from RDS.
• Reads a text file of gene names.
• Produces and saves UMAP and spatial plots.
• If no gene list is provided, plotting is skipped.
Key functions

• Generates FeaturePlot on UMAP for each specified gene.
• Creates ImageFeaturePlot to overlay gene expression on spatial coordinates.

To ensure package version stability and compatibility, we provide users with Docker and Singularity container manifests for the 2 latter pipeline components. This further allows users to build and run NNclinSSOAP in cloud-based containers.

In practice, the “Methods” section in this paper outlines a demo case that enables users to familiarize themselves with NNclinSSOAP and convert raw sc/snRNA-seq and Spatial Transcriptomics data into spatially resolved tissue maps with gene expression and cell-type annotations, through a small number of configuration changes and straightforward execution commands. The steps are divided into 2 procedures (Procedures 1 and 2) for sn/scRNA-seq and Spatial Transcriptomics, respectively. These support local execution on the demo datasets and outline the possibility of deployment on cloud infrastructure for larger datasets.

**Fig. 2.**
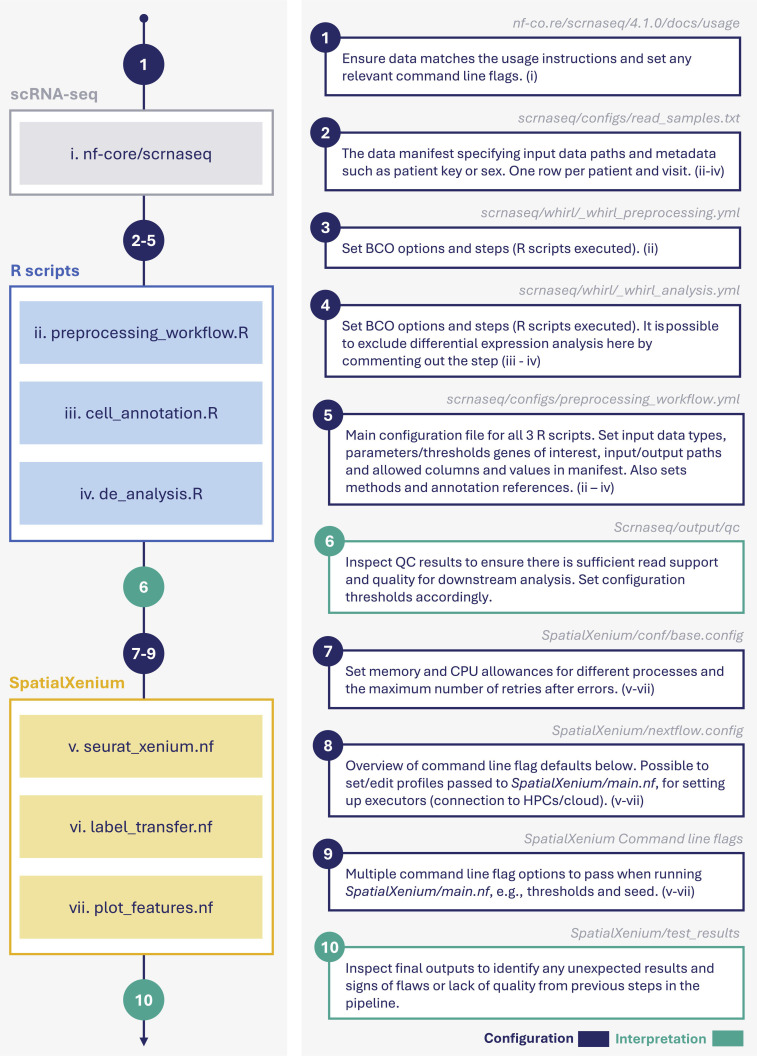
Overview of user-configurable inputs and review points in NNclinSSOAP. The left panel shows the main workflow components and scripts/modules; the right panel summarizes the configuration files, command-line options, and QC/output review points. Numbers link each configuration or review point to the relevant workflow step. Paths are shown relative to the NNclinSSOAP repository root.

For usage other than the demo case, several configurable settings are available. These are shown at a high level in Fig. 2. Importantly, in a clinical setting, the configurations should be set and documented before the pipeline is executed. To see all the options available, please refer to the documentation available in the repository.

### GxP-readiness

In this manuscript, GxP-readiness refers to the ability of a computational workflow to be deployed within a regulated clinical-research environment in a way that supports data integrity, traceability, reproducibility, auditability, and controlled change management. The term does not imply that the pipeline alone makes an analysis fully GxP compliant. Rather, NNclinSSOAP provides pipeline-level controls that can be combined with an institution’s validated infrastructure, standard operating procedures, access controls, and documentation practices. Relevant expectations for clinical computerized systems and analysis planning include principles described in ICH E6(R3) [11], ICH E9 [12], and FDA 21 CFR Part 11 [13].

NNclinSSOAP implements these pipeline-level controls through a modular architecture, containerized execution environments, and automatic provenance capture. The whirl package provides the functionality of executing scripts in batch and simultaneously getting a log from the individual execution [14]. Nextflow, an open-source workflow management system [15], allows each workflow step to be run as a specified module with captured parameters and process metadata, while containers help control software versions and dependencies. The entire workflow is documented automatically in BioCompute Objects (BCOs). They document the intended purpose, inputs, software and version identity, parameter settings, execution details, provenance, and output locations of an analysis, providing a standardized record that can be reviewed, repeated, and archived [16,17]. BioCompute is an FDA-supported standard for regulatory submissions, and can be used during regulated activities and communication with the agency [18].

In clinical use, the GxP-ready pipeline is intended to be executed after the analysis has been specified in the clinical study analysis plan. Input data, configuration files, and output objects are treated as controlled analysis artifacts. Sample metadata and QC thresholds are defined before execution where possible, the run is executed in a validated or otherwise qualified compute environment, and generated reports, logs, BCOs, and Seurat objects are retained as evidence of what was run.

In our implementation, clinical data storage and access are handled by a compliant Amazon Web Services (AWS) storage service with separate input and output locations and role-based access. For data processing, analyses were performed within a dedicated, GxP-validated compute environment developed in-house as part of the Novo Nordisk secure analytics platform. This environment operates within a cloud-based infrastructure to provide both scalability and regulatory compliance throughout the data life cycle. The flow of inputs and outputs and the environments used is shown in Fig. 1. Other institutions can reuse the open-source code, containers, and provenance mechanisms that NNclinSSOAP provides, connecting the workflow to their own validated compute, storage, access-control, retention, audit-trail, and electronic-signature processes.

The scope of readiness is therefore layered. Elements that transfer directly with the pipeline include modular workflow definitions, container manifests, configurable analysis parameters, BCO generation, execution logs, and standard outputs for review. Elements that remain institution-specific include formal software validation or verification, training, role-based review and approval of BCOs, controlled versioning and change management of the validated pipeline, secure data retention, audit trails, and any electronic-signature practices. This distinction is important because BioCompute primarily defines what is documented. It does not replace local validation, governance, or standard operating procedures required for full GxP compliance.

Within the pipeline, GxP-readiness is also reflected in how analysis decisions are exposed rather than hidden. QC filters and label-transfer probability thresholds are explicit user-configurable parameters. Cells failing QC are excluded from downstream analysis according to documented thresholds, and low-probability labels are not silently converted into confident cell types. Instead, they are flagged as “Ambiguous” in the labeled Seurat metadata and visualizations and are accompanied by prediction files. This allows downstream users and reviewers to distinguish confident annotations from those requiring caution or manual review.

### Applications

NNclinSSOAP was specifically developed for clinical trials incorporating transcriptomics as study endpoints, including sc/snRNA-seq and Spatial Xenium. As a pioneering framework in the pharmaceutical industry, particularly for cardiometabolic diseases, NNclinSSOAP has already been used in 2 mechanism-of-action trials. The first trial, focused on Alzheimer’s disease, incorporated scRNA-seq data as a primary endpoint [19], and therefore utilized only Procedure 1, which is tailored for single-cell transcriptomics. In contrast, the second trial, a clinical study in kidney disease [20,21], employed both Procedures 1 and 2 as it included both snRNA-seq and Xenium Spatial Transcriptomics data.

By enabling rapid and GxP-aligned data processing, this workflow sets a new standard for transcriptomic analysis in clinical trials. Looking ahead, it is broadly applicable to trials in other therapeutic areas, provided that compatible input data are available. Thanks to Nextflow’s modular architecture, the workflow can be easily adapted to accommodate additional analytical modules in the future.

Notably, the SpatialXenium Nextflow pipeline described in this paper is publicly available as part of the repository and can be integrated into new or existing Nextflow workflows, independently of the broader workflow described in the paper.

### Comparison to other methods

There are numerous tools and packages available to facilitate sc/snRNA Transcriptomics data preprocessing [22]. For sc/snRNA-seq, we used the established nf-core pipeline scrnaseq as part of Procedure 1. Beyond the initial preprocessing steps, downstream processing and analysis of sc/snRNA-seq data commonly leverage widely used packages such as Seurat [23] that we use as part of our R library. Our SpatialXenium pipeline is wrapped around the Seurat suite relevant for Spatial Transcriptomics data QC, preprocessing, and analytics.

Integrated platforms for combining single-cell and Spatial Transcriptomics exist [24], but not as workflows aligned with GxP requirements. The purpose of this section is to contextualize NNclinSSOAP rather than to provide a formal benchmarking study. NNclinSSOAP builds on established community methods and combines them into a documented, containerized, and provenance-aware workflow for regulated clinical research settings. The points below therefore focus on workflow structure, traceability, reproducibility, and deployment considerations. Six main features make NNclinSSOAP different to other methods:

1. Modularity. NNclinSSOAP not only makes it possible to run the underlying sc/snRNA-seq and SpatialXenium workflows separately, but the workflows themselves build on Nextflow modules. This simplifies both using the workflows and adapting them to future needs as well as making them scalable, a key factor in data heavy settings.
2. GxP-readiness. Using Nextflow for building our workflows enables NNclinSSOAP to be both scalable and reproducible. Combined with BCO-based provenance capture, this supports selected GxP-relevant expectations, particularly traceability, reproducibility, and reviewability. It allows users to connect to a compute environment of choice, without compromising reproducibility, enabling analysis of data volumes at a much greater scale than in the demo case described in this paper. Full GxP compliance ultimately depends on deployment within an appropriate governed environment.
3. The SpatialXenium pipeline. There is a publicly available pipeline, nf-core/spatialxe, currently under development. It is a flexible image- and coordinate-based reanalysis framework, focused on controlling and improving Xenium data quality through multiple segmentation and spatial-data methods. SpatialXenium differs from this, instead focusing on postprocessing and analysis of Xenium data. It provides users a fully specified, protocol-locked, and easily reproducible end-to-end pipeline, implementing Spatial Transcriptomics analysis from Seurat for Xenium data.
4. We provide an R library capable of processing outputs generated by nf-core/scrnaseq, supporting standard Seurat workflows and downstream annotation. These scripts offer flexibility, incorporating tools such as Harmony [25] and RPCA [26] for multisample integration. For cell-type annotation, automated approaches like Azimuth [27] or SingleR [28] are supported, and user-defined reference datasets can be added. For differential treatment effect analysis, we use a pseudobulk [29] approach by aggregating counts per sample and cell type, followed by statistical assessment using edgeR [30], which enables robust testing of group-level differences while accounting for biological variability. Alternatively, other statistical methods can be integrated as needed. An overview of this workflow is illustrated in Fig. 1.
5. By leveraging the whirl package [14] to execute the R library, the pipeline produces a BCO along with multiple log files—one corresponding to each R script. The package offers the flexibility to modify certain domains within the BCO and to select specific scripts to run as needed.
6. The R environment required for running these procedures is provided as a Docker container, ensuring that all necessary packages and dependencies are included and eliminating compatibility issues for the user. This approach allows the entire environment to be reliably loaded and the workflow to be executed as intended. However, one limitation is that package versions within the container may become outdated as new releases are made. Users also have the flexibility to set up and use their own custom environments if preferred.

## Experimental Design

### Expertise needed for implementation and interpretation

NNclinSSOAP was developed to be used by computational biologists, producing results (e.g., tables and figures) that can be interpreted and understood by medical or biological experts without a data science background. For users following the procedures step-by-step, no expertise is needed and none of the topics below need to be considered. However, some familiarity with single-cell transcriptomics and relevant training in statistical analysis of high-dimensional omics data and running analytics scripts is advisable for a smooth experience and ability to understand the outputs scientifically.

If the workflow outlined in this paper is adapted to another dataset different from the demo, carefully consider the points below. It is also recommended to read the extensive information and instructions provided in the code repository *README.md* and .md files in */docs*. For this, a broad understanding of R, Nextflow, cloud computing, containerization, and single-cell transcriptomics is needed.

### Sampling strategy-dependent analysis

The datasets used in Procedure 2 contain a single sample each, one with healthy kidney tissue and the other with cancerous kidney tissue. This case facilitates analysis from the perspective of seeing differences in cell types and gene expression. Depending on a dataset’s sampling strategy, experimental design, and analysis plans, as often preregistered in a clinical trial setting, other relevant analyses and visualizations are possible, e.g., mitochondrial genes, cell cycle genes, biological sex-related genes, batch effect contribution from metadata, etc. For samples taken from the same subject at multiple time points (longitudinal data), it is possible to analyze changes over time. For samples taken at a single time point for multiple subjects (cross-sectional data), it is possible to analyze the homo-/heterogeneity within a population. Combining these 2 strategies creates a clinical trial scenario where NNclinSSOAP can be used to analyze subjects’ tissue characteristics over time as a response to treatment, comparing to a placebo group or among groups at a single time point for disease understanding.

### Optional settings and configurations

Beyond the default settings and basic configurations required to run the demo case in the “Methods” section, NNclinSSOAP can be customized through various command line flags and configuration files. Figure 2 provides a high-level overview of these options.

For the sc/snRNA-seq workflow, users can customize the metadata and configuration files to use with their own data, according to the nf-core/scrnaseq documentation: https://nf-co.re/scrnaseq/4.0.0/docs/usage/. For improved version control and streamlined collaboration, it is highly recommended to maintain a dedicated Git repository containing all related metadata and configuration files.

**Fig. 3.**
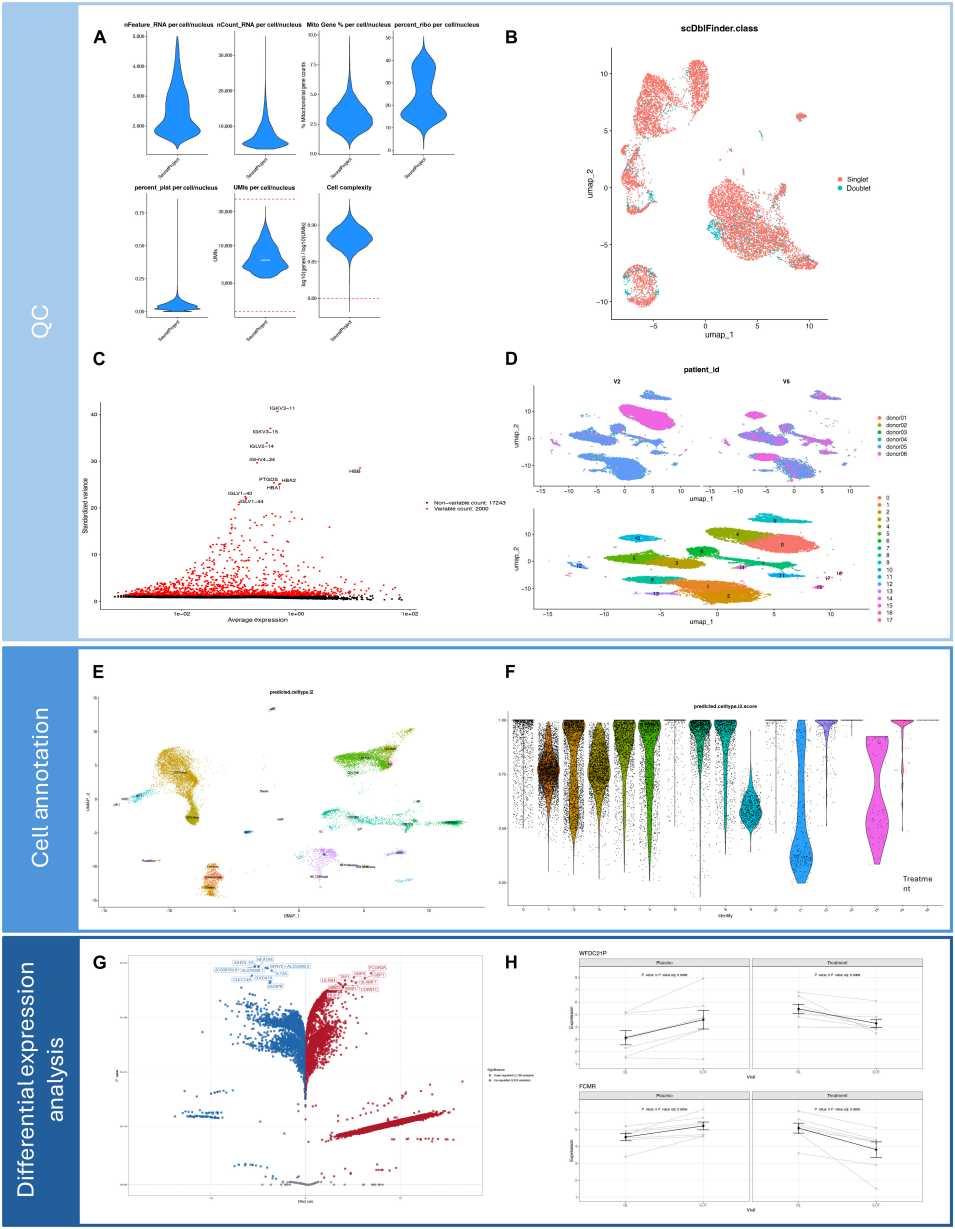
Summary of sc/snRNA-seq data analysis workflow and results. (A) QC violin plots displaying the distribution of nFeature_RNA, nCount_RNA, mitochondrial percentage, ribosomal percentage, platelet percentage, UMI counts, and cell complexity per cell. (B) UMAP projection highlighting doublet detection results. (C) Identification of top highly variable genes used for downstream analysis. (D) Integration UMAP visualizations, stratified by patient ID (top) and by cluster (bottom). (E) UMAP embedding with annotated cell types. (F) Violin plots showing prediction scores for cell-type assignment. (G) Volcano plot displaying differential expression analysis results illustrating treatment effects. (H) Line plot illustrating the averaged expression levels of the top differentially expressed genes across treatment groups.

Once the nf-core/scrnaseq pipeline has completed, NNclinSSOAP provides users with tools to further analyze and explore their data. Specifically, 3 main R scripts are included for downstream analysis (see Fig. 3), each designed with flexibility in both parameters and methods. To customize or modify the pipeline execution, users can edit the associated config file *preprocessing_workflow.yml*. These options provide users with extensive flexibility for preprocessing, integrating, and annotating single-cell data within the NNclinSSOAP framework. Note that the RPCA integration method is computationally intensive and may not be suitable for local execution.

To load their own data into the pipeline, users must modify the *configs/read_samples.txt* file, ensuring it accurately lists each sample along with its corresponding metadata. Column names as well as visit and treatment arm values can be set from *configs/preprocessing_workflow.yml*. All columns are intended to populate the Seurat object’s metadata, but if your dataset lacks certain variables (arm and visit), populate them with an NA and scripts 1 (*preprocessing_workflow.R*) and 2 (*cell_annotation.R*) will still function, allowing you to generate an integrated and annotated Seurat object.

Script 3 (*de_analysis.R*), designed for differential gene expression analysis in clinical settings to assess treatment response, specifically calculates treatment effects, which assumes the data are longitudinal, with 2 time points (a baseline and follow-up visit) and 2 distinct treatment arms (e.g., drug A and placebo), as is the case in the demo data (see Fig. 3). If your data are not longitudinal, this script can be excluded.

Since the pipeline is run using the whirl wrapper, users can also adjust the *whirl/_whirl_preprocessing.yml* and *whirl/_whirl_analysis.yml* config files to edit BCO domains or exclude scripts, such as *de_analysis.R*, from execution as needed.

For SpatialXenium, the genes shown in the UMAP and spatial feature plots are read from *assets/genes.txt* in the Spatial Transcriptomics workflow. By default, this file contains the genes needed to reproduce our results on the demo case data. Users should adapt this file if using other datasets, to reflect their genes of interest. Multiple command line options control which cells and genes are included in the UMAP and spatial feature plots, and how strict the filtering is.

### Quality control

**Fig. 4.**
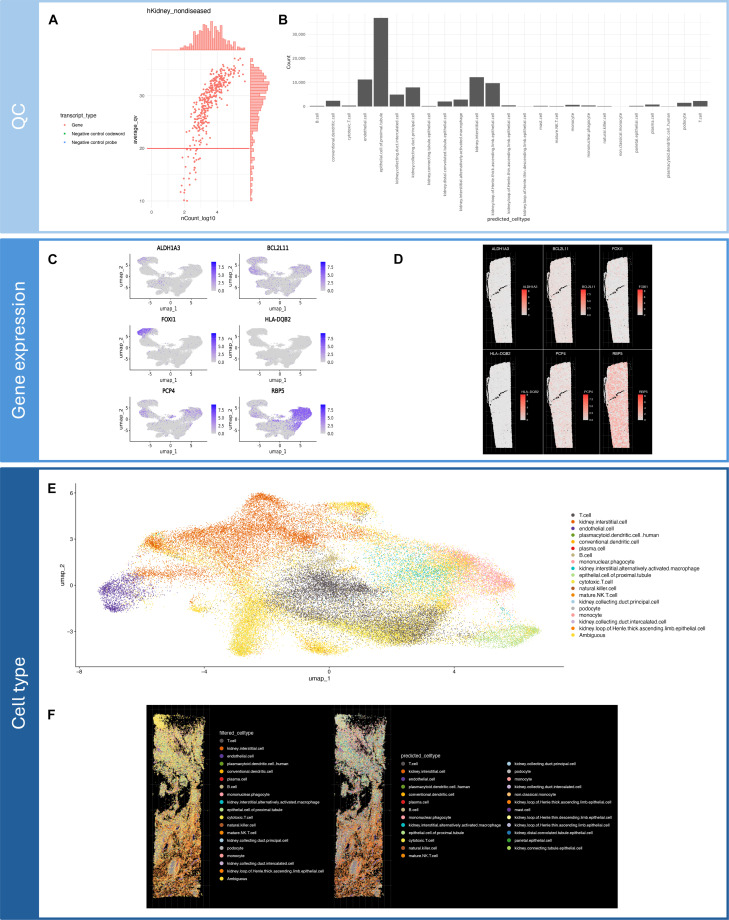
Visualizations generated by the Spatial Transcriptomics pipeline relating to Quality Control, Gene Expression and Label Transfer. (A) Scatter plot of the QV score vs. the counts of all genes, with histograms demonstrating the distribution of genes in each axis. (B) Histogram showing the count of each predicted cell type. (C) UMAPs highlighting the density of each gene (gradient of purple–blue) from genes.txt in the UMAP space containing all genes (gray area). (D) Tissue maps highlighting the location and level of gene expression (gradient of red) for each gene in genes.txt. (E) UMAP showing all the cell types, set to “Ambiguous” below the probability threshold specified. (F) Tissue maps with predicted cell types filtered by the specified probability threshold highlighted (left) and all predicted cell types (right).

QC thresholds should be reviewed in the context of each dataset and, where applicable, defined before downstream biological or clinical interpretation. For Xenium data, the relationship between read counts, gene detection, and QV score provides a practical check of assay quality (see Fig. 4). A high-quality dataset is expected to retain most informative cells and genes after filtering, with low-quality or control-probe-rich observations limited to a minority of the data. In contrast, a problematic dataset may show a large fraction of observations with low read counts, low QV scores, unusually high read-count outliers, or high control-probe proportions. The selected thresholds are captured in configuration files and execution outputs (see Fig. 2), supporting reproducibility and review of the filtering decisions.

### Seurat Spatial Transcriptomics visualization options

The tissue visualizations overlaid with gene expression and cell types generated from running SpatialXenium utilize the Seurat v5 package’s functions ImageDimPlot()and ImageFeaturePlot(). These offer a wide range of options beyond what is covered by default in SpatialXenium, enabling extensive control over plot aesthetics and content, including color scales, transparency, cell border properties, molecule overlays, coexpression blending for pairs of genes and grouping cell types. For advanced users wanting to tailor SpatialXenium visualizations to their specific biological questions, ImageDimPlot() is called from *label_transfers.r* and ImageFeaturePlot() is called from *plot_features.r*. We strongly recommend familiarity with R, Seurat, and Nextflow for doing this, as making changes may result in errors downstream.

A vignette outlining the visualization possibilities can be accessed here: https://satijalab.org/seurat/articles/seurat5_spatial_vignette_2.html

### Open-source strategy

Given the variability between clinical study designs and datasets, we chose to make NNclinSSOAP open-source, allowing users to adapt the pipeline beyond what is possible through the config and flag settings. This also allows users to use specific modules, such as SpatialXenium, instead of the entire pipeline. From a development and maintenance point of view, the open-source strategy invites users to contribute to the project as well as report any issues they encounter.

### Compute environment

NNclinSSOAP was optimized to run on high-performance computing environments. For running the minimal demo case described in this paper, the hardware requirements are basic enough to use a laptop. However, this is unlikely to be the case if attempting to analyze larger datasets, such as the benchmark datasets listed in the “Data Availability” section. Benchmarking results from running the SpatialXenium workflow on these in our cloud environment are listed in Table 1.

**Table 1.** Runtime and memory usage examples for SpatialXenium. Computational requirements and duration are shown for public Xenium datasets run in the authors’ cloud environment. These values are intended to guide compute planning across dataset sizes and are not presented as a formal performance benchmark against other tools.

Dataset	Cell count	Xenium input size	Output size	Duration	Max RAM required
Kidney nondiseased	97,560	9.4 GB	695 MB	13 min 37 s	11.9 GB
Kidney cancer (PRCC)	56,510	6.4 GB	449 MB	10 min 17 s	10.2GB
Human skin melanoma	106,980	12 GB	2.3 GB	16 min 18 s	14.8 GB
Pancreatic cancer	190,965	7.8 GB	3.1 GB	25 min 3 s	18.1 GB
Cervical cancer 5k	840,387	69 GB	6.6 GB	4 h 38 min 33 s	75.7 GB

Memory and CPU allowances for different processes in the SpatialXenium pipeline can be customized in *conf/base.config*. The pipeline also allows usage of alternative containerization platforms to Docker, such as Singularity, through the -profile Nextflow argument. In addition to this, Nextflow has options for connecting executors such as HPC/cloud computing services by editing *nextflow.config*, further explained here: https://www.nextflow.io/docs/latest/executor.html

## Materials and Equipment Setup

To meet all prerequisites for following the “Methods” section, ensure that all the requirements in this section are met, and any given instructions are followed.

### Hardware requirements

• Any desktop workstation or laptop with an internet connection is sufficient. The procedures were tested on a MacBook Pro (MacOS Tahoe 26.1) with a 12-Core central processing unit (CPU) and 24 GB of random-access memory (RAM). For minimal performance, we recommend using 4 CPUs with at least 18 GB of RAM for analyses.

### Software requirements

• Operating system: Linux, Windows, or MacOS
• IDE: An integrated development environment. We recommend Visual Studio Code, which can be accessed at https://code.visualstudio.com/download.
• Docker desktop: A platform for docker container management, which can be accessed at https://docs.docker.com/desktop/.
• Nextflow: A workflow system for creating scalable, portable, and reproducible workflows, which can be accessed at https://www.nextflow.io/docs/latest/install.html. The version used here is 25.10.0.

### Installation of Docker desktop

Install Docker desktop from the official website: https://www.docker.com/products/docker-desktop/. Once downloaded, open the application and go to Settings > Resources and change the CPU setting to a minimum of 4 and the memory setting to a minimum of 18 GB.

### Installation of Nextflow

Install Nextflow by following the instructions on the official website: https://www.nextflow.io/docs/latest/install.html. You can check if Nextflow is correctly downloaded and available by typing the following in your terminal: nextflow info

### Cloning the repository

Open a terminal and navigate to the directory you want to place the repository in, then run:

> git clone https://github.com/NovoNordisk-OpenSource/nnclinssoap.git

### Downloading the demo case data

Inside your IDE of choice, open the cloned repository. In the terminal, run the following: > bash ./setup_test_data.sh

You should see the download start in the terminal. Once finished (approximately 1 h), the data are found in the directories *scrnaseq/test_data* and *SpatialXenium/test_data*.

### Building the docker images

Start by making sure Docker desktop is open. Then, inside your IDE of choice, open the cloned repository. In the terminal, run the following: > cd docker && bash ./build_all_images.sh -v docker

Build progress is shown in the terminal and Docker desktop. Building all images takes around 3 to 6 h.

## Methods

This section describes how to use NNclinSSOAP with the provided demo case data, ensuring an easy-to-follow introduction to usage. For users who wish to customize their analysis, use other data, or run the pipeline in an HPC, the repository is open-source and contains more detailed documentation.

We divide the methods in 2 procedures, which can be performed sequentially or independently. Procedure 1 describes the preparation and steps needed for running the sc/snRNA-seq workflow. Procedure 2 covers the preparation and steps needed for running the SpatialXenium workflow, with an option to incorporate the annotated Seurat object generated from Procedure 1 as input for cell label transfer.

If problems arise while following the procedure, please refer to the “Troubleshooting” section.

### Procedure 1: Sc/snRNA-seq ~ 1h

**Run nf-core/scrnaseq preprocessing (test execution) ~ 10 min**

1. Open the nnclinssoap repository in your IDE.
2. In the terminal, run the following:


            > nextflow run nf-core/scrnaseq -profile test,docker --outdir ./results

3. Once the pipeline has run, the output files will be placed under the output folder */results*.

**Run the pipeline ~ 50 min**

**Note:** Docker desktop must be open.

4. Run counts matrix processing into Seurat object. In the terminal, run the following:

 > cd scrnaseq > bash ./run_preprocessing_containerized.sh

5. Run cell annotation and differential expression analysis. In the terminal, run the following:

 > bash ./run_analysis_containerized.sh

### Procedure 2: SpatialXenium ~ 15 min

**Set configurations ~ 3 min**

1. Open the nnclinssoap repository in your IDE.
2. Open *SpatialXenium/conf/base.config* and check if any of withLabel:process_single, withLabel:process_low, withLabel:process_medium or withLabel:process_high have a CPU or memory setting exceeding your available resources and the settings in Docker Desktop. If so, change them to be within that range.
3. (Optional) Open *SpatialXenium/conf/test.config* and change reference_rds to the path of the annotated Seurat object created in Procedure 1, step 5 (should be *scrnaseq/output/seurat_object.rds).*
In the same file, change single_cell_label_col to “predicted.celltype.l1”.

**Run the pipeline ~ 12 min**

**Note:** Docker desktop must be open.

4. In the terminal, run the following:

> cd SpatialXenium> nextflow run main.nf -profile test,docker --outdir test_results

5. Once the pipeline has run, the output files will be placed under the output folder */test_results*.

## Troubleshooting

For general troubleshooting, there are shell scripts designed to validate that the prerequisites for running NNclinSSOAP are in place, located in the directory */tests*. Run them from the terminal and read through the resulting logs. The scripts are described below:

• *test_containers.sh*: Container testing Suite. Tests all built containers for correct functionality.
• *spatialxenium_preflight_check.sh*: Preflight check for SpatialXenium pipeline testing. Verifies all dependencies and setup before running tests.

In case running these scripts does not indicate where your issue lies, refer to Table 2 for further troubleshooting related to the specific steps in the “Methods” section.

**Table 2.** Step-specific troubleshooting. A collection of problems related to the different steps in the “Methods” section, with possible reasons and solutions.

Step	Problem	Possible reason	Solution
General troubleshooting			
	Error when running a shell script	Bash version to low	Ensure that the latest bash version is installed and verify it in the terminal you run the scripts from with: bash --version
	nextflow: command not found	Nextflow binary file not on PATH	Locate Nextflow binary (run: which nextflow) and add it to PATH.
	Data not downloaded	Download was interrupted	Remove any *test_data/* folders inside *scrnaseq/* and *SpatialXenium/* and re-run *setup_test_data.sh*.
Troubleshooting for procedure 1 (sc/snRNA-seq)			
2	Pulling nf-core/scrnaseq ... WARN: Cannot read project manifest -- Cause: PKIX path building failed: sun.security.provider.certpath.SunCertPathBuilderException: unable to find valid certification path to requested target	Java Virtual Machine (JVM) does not have access to the server certificate or its certificate chain	1. Fix java specific certificates 2. Clone the nf-core pipeline repository and call it locally
4	Error in Seurat::Read10X_h5(filename = paste0(sample_path, “/sample_filtered_feature_bc_matrix.h5”)	The data are not in the right path	Check the downloaded data is in the right path *nnclinsoap/scrnaseq/test_data/*
Troubleshooting for procedure 2 (SpatialXenium)			
4	Memory issues (error codes 104, 130–145)	The memory settings in the workflow exceeded those made available	i. Check that none of the memory variables in step 2 exceed what is available on your computer. If so, assign them a lower value. ii. Check that your docker containers’ memory allocation match those in (i) as described in the Docker Desktop installation.

## Expected Results

The result of following both procedures is a transformation of raw sc/snRNA-seq data into an integrated and annotated Seurat object, and raw Xenium data into a spatially resolved cell object and map. This can aid in answering clinical questions, giving the user a deeper understanding of the analyzed tissues both at a cellular expression and a structural level, as is the case in the demo case data in this paper, highlighting differences between healthy and cancer kidney tissue.

When the procedures are executed with the supplied demo data using the specified software versions, parameters and settings, deterministic tables and files are expected to be reproducible. Stochastic visualization steps such as UMAP may show minor layout differences while preserving the same biological topology and interpretation. If the paper is followed using a personal dataset, the same types of outputs are expected, but results will differ according to the input data.

### Sc/snRNA-seq (procedure 1)

Executing the sc/snRNA-seq workflow (Procedure 1, step 2) generates an nf-core results folder. The demo case dataset has been run with the cellranger aligner [31], so the results are the standard cellranger outputs. The special addition is the BCO object that has been generated since the plugin is included when running the nextflow pipeline.

In the second part of the workflow (Procedure 1, steps 4 and 5), each script produces a variety of plots and output files, which are organized into dedicated folders—see Box 2 for a summary of the results file structure.

• Quality Control (QC): QC plots document the standard Seurat workflow, starting from reading the counts matrix and progressing through to the creation of the final Seurat object (see Fig. 3A to D). A separate folder is generated for each sample’s QC outputs, while the integrated Seurat object is stored in the parent directory.
• Cell Annotation: This step generates visualizations such as UMAPs displaying annotated clusters and violin plot displaying the annotation scores, along with an updated Seurat object that contains cell type assignments (see Fig. 3E and F).
• Differential Expression analysis: For each cell type, the pipeline outputs a normalized counts CSV file, a CSV file with differential expression results, and a volcano and line plot summarizing key findings (see Fig. 3G and H).

Box 2. Example file structure from Procedure 1.5

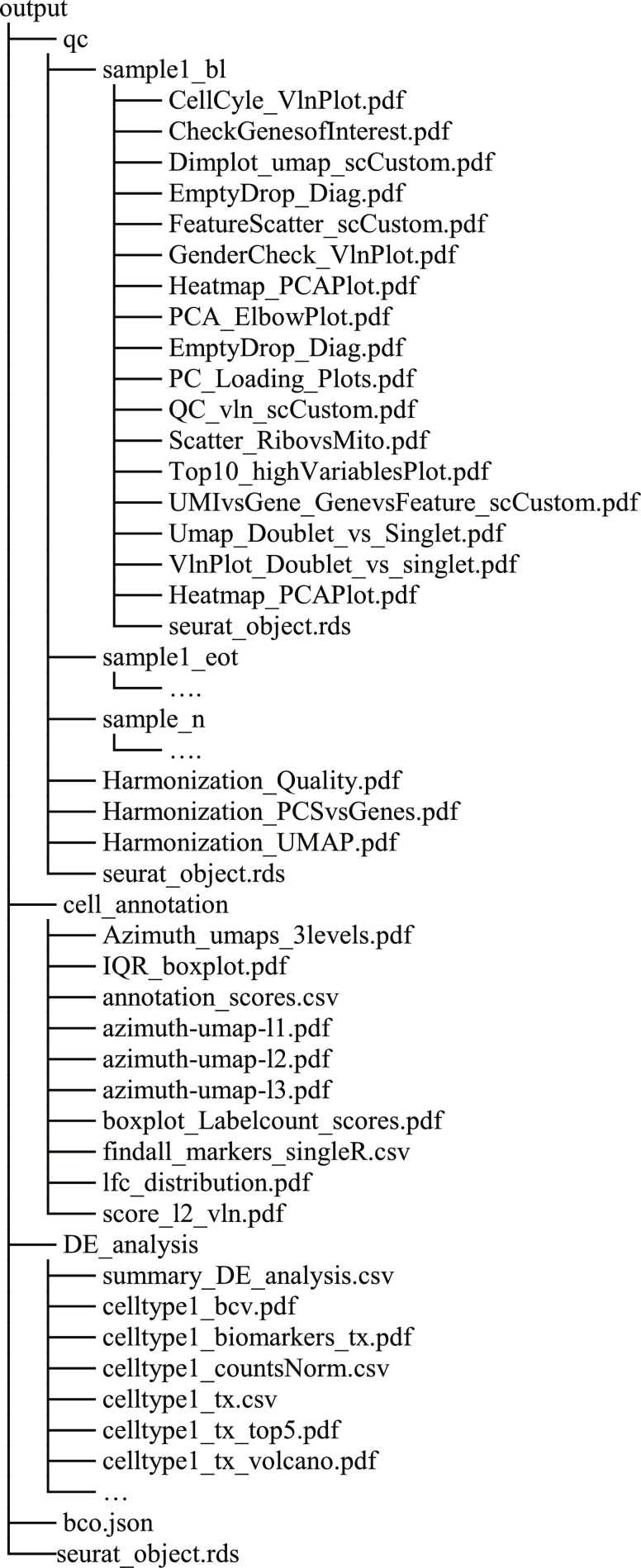

### SpatialXenium (procedure 2)

Executing the SpatialXenium workflow (Procedure 2, step 4) produces several key outputs that can be used for clinical interpretation of the tissues analyzed, shown in Fig. 4. By using Nextflow, reports covering errors, timing and resource usage have automatically been created. An example results file structure is shown in Box 3. To aid with technical and clinical interpretation, key outputs from Quality Control (QC), gene expression and cell type analysis are described in further detail below:

• Quality Control: The QC plots (see Fig. 4A and B) summarize how many genes and reads are captured per cell and how these values are distributed across the dataset. This allows users to identify low-quality cells, technical outliers, or regions with suboptimal capture efficiency before drawing any biological or clinical conclusion, ensuring that downstream patterns (e.g. “immune poor” areas) are not artifacts of poor data quality.
• Gene Expression: The gene expression visualizations (see Fig. 4C and D) show how the selected markers from *assets/genes.txt* are distributed both in UMAP space and across the tissue. Clinically, these plots help confirm that canonical markers (for example, tumor, immune, or stromal genes) are expressed in the expected tissue compartments and spatial niches, supporting interpretations such as tumor–stroma interactions, immune infiltration patterns, or localization of specific disease-relevant pathways.
• Cell Type: The cell-type plots (see Fig. 4E and F) resulting from the label transfer display the predicted cell types in UMAP space projected back onto the tissue, with cells below the chosen probability threshold set to “Ambiguous”. This makes it clear which assignments are robust enough to support biological or clinical interpretation, which should be treated with caution. In a clinical context, these maps can be used to explore how specific cell types (e.g., cytotoxic T cells, fibroblasts, and endothelial cells) are spatially organized within lesions, margins, or normal tissue, and how this architecture might relate to disease severity or therapeutic response.

Box 3. Example file structure. Full sample file structure only shown for sample 1 for simplicity.

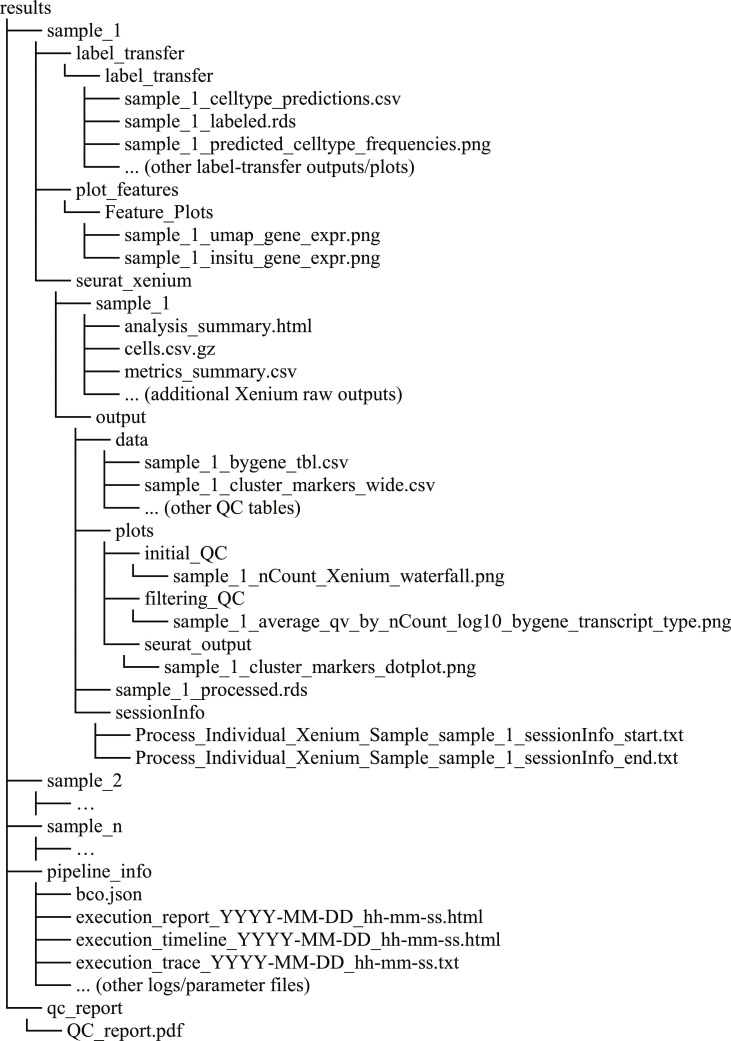

## Conclusion

NNclinSSOAP offers a GxP-ready framework for scalable processing and analysis of single-cell and Spatial Transcriptomics data for clinical research and trials. Its modular design and automated provenance capture ensure transparent, reproducible, auditable, and fully traceable analyses suitable for regulated environments, e.g., clinical trials. Together, these features provide a practical, unique, and extensible foundation for deploying multimodal transcriptomics across mechanistic studies and clinical trials.

## Data Availability

Requests for further information and resources should be directed to and will be fulfilled by the lead contact, V.D. (VVDA@novonordisk.com). **Code:** The NNclinSSOAP repository is available here: https://github.com/NovoNordisk-OpenSource/nnclinssoap. The manuscript-associated version of NNclinSSOAP is be archived on Zenodo and cited using a persistent DOI here: https://doi.org/10.5281/zenodo.20783108. This archived release should be used to reproduce the analyses described in this paper, while the GitHub repository will remain the live location for future development, issue tracking, and community contributions. **Data used in the “Methods” section:** All data used in the Methods section are available from the links listed below:• scRNA-seq h5 matrixes: https://zenodo.org/records/18173543. • Xenium Kidney (both nondiseased and cancer): https://www.10xgenomics.com/datasets/human-kidney-preview-data-xenium-human-multi-tissue-and-cancer-panel-1-standard. • Human kidney cell annotation reference: https://zenodo.org/records/10694842. **Benchmark datasets:** Datasets that can be used for benchmarking purposes are available from the links listed below:• Human Skin Melanoma: https://www.10xgenomics.com/datasets/human-skin-preview-data-xenium-human-skin-gene-expression-panel-1-standard . • Pancreatic Cancer: https://www.10xgenomics.com/datasets/pancreatic-cancer-with-xenium-human-multi-tissue-and-cancer-panel-1-standard. • Cervical Cancer 5k: https://www.10xgenomics.com/datasets/xenium-prime-ffpe-human-cervical-cancer. **Fastq dataset:** Raw data for creating the scRNA-seq h5 matrixes using nf-core/scrnaseq are available from the links listed below:• Universal 5′ dataset: https://www.10xgenomics.com/datasets/5-hashing-example-with-tabs-2-standard.
